# Predicting Mortality in Intensive Care Unit Patients With Heart Failure Using an Interpretable Machine Learning Model: Retrospective Cohort Study

**DOI:** 10.2196/38082

**Published:** 2022-08-09

**Authors:** Jili Li, Siru Liu, Yundi Hu, Lingfeng Zhu, Yujia Mao, Jialin Liu

**Affiliations:** 1 West China School of Medicine Sichuan University Chengdu China; 2 Department of Biomedical Informatics Vanderbilt University Medical Center Nashville, TN United States; 3 School of Data Science Fudan University Shanghai China; 4 Department of Computer Science Sichuan University Chengdu China; 5 Department of Medical Informatics West China Hospital Sichuan University Chengdu China

**Keywords:** heart failure, mortality, intensive care unit, prediction, XGBoost, SHAP, SHapley Additive exPlanation

## Abstract

**Background:**

Heart failure (HF) is a common disease and a major public health problem. HF mortality prediction is critical for developing individualized prevention and treatment plans. However, due to their lack of interpretability, most HF mortality prediction models have not yet reached clinical practice.

**Objective:**

We aimed to develop an interpretable model to predict the mortality risk for patients with HF in intensive care units (ICUs) and used the SHapley Additive exPlanation (SHAP) method to explain the extreme gradient boosting (XGBoost) model and explore prognostic factors for HF.

**Methods:**

In this retrospective cohort study, we achieved model development and performance comparison on the eICU Collaborative Research Database (eICU-CRD). We extracted data during the first 24 hours of each ICU admission, and the data set was randomly divided, with 70% used for model training and 30% used for model validation. The prediction performance of the XGBoost model was compared with three other machine learning models by the area under the curve. We used the SHAP method to explain the XGBoost model.

**Results:**

A total of 2798 eligible patients with HF were included in the final cohort for this study. The observed in-hospital mortality of patients with HF was 9.97%. Comparatively, the XGBoost model had the highest predictive performance among four models with an area under the curve (AUC) of 0.824 (95% CI 0.7766-0.8708), whereas support vector machine had the poorest generalization ability (AUC=0.701, 95% CI 0.6433-0.7582). The decision curve showed that the net benefit of the XGBoost model surpassed those of other machine learning models at 10%~28% threshold probabilities. The SHAP method reveals the top 20 predictors of HF according to the importance ranking, and the average of the blood urea nitrogen was recognized as the most important predictor variable.

**Conclusions:**

The interpretable predictive model helps physicians more accurately predict the mortality risk in ICU patients with HF, and therefore, provides better treatment plans and optimal resource allocation for their patients. In addition, the interpretable framework can increase the transparency of the model and facilitate understanding the reliability of the predictive model for the physicians.

## Introduction

Heart failure (HF), the terminal phase of many cardiovascular disorders, is a major health care issue with an approximate prevalence of 26 million worldwide and more than 1 million hospital admissions annually in both the United States and Europe [1]. Projections show that by 2030 over 8 million Americans will have HF, leading to an increase of 46% from 2012 [2]. Each year, HF costs an estimated US $108 billion, constituting 2% of the health care budget globally, and it is predicted to continue to rise, yet half of it is possibly avoidable [3]. As COVID-19 continues to spread across the world, HF, a severe complication, is associated with poor outcome and death from COVID-19 [4,5].

The critically ill patients in intensive care units (ICUs) demand intensive care services and highly qualified multidisciplinary assistance [6]. Although ICU plays an integral role in maintaining patients’ life, this also implies the workforce shortage, limited medical resources, and heavy economic burden [7]. Therefore, early hospital mortality detection for patients is necessary and may assist in delivering proper care and providing clinical decision support [8].

In recent years, artificial intelligence has been widely used to explore the early warning predictors of many diseases. Given the inherent powerful feature of capturing the nonlinear relationships with machine learning algorithms, more researchers advocate the use of new prediction models based on machine learning to support appropriate treatment for patients rather than traditional illness severity classification systems such as SOFA, APACHE II, or SAPS II [9-11]. Although a large number of predictive models have shown promising performance in research, the evidence for their application in clinical setting and interpretable risk prediction models to aid disease prognosis are still limited [12-15].

The purpose of our study is to develop an interpretable model to predict the risk mortality for patients with HF in the ICU, using the free and open critical care database—the eICU Collaborative Research Database (eICU-CRD). In addition, the SHapley Additive exPlanations (SHAP) method is used to explain the extreme gradient boosting (ie, XGBoost) model and explore prognostic factors for HF.

## Methods

### Data Source

The eICU-CRD (version 2.0) is a publicly available multicenter database [16], containing deidentified data associated with over 200,000 admissions to ICUs at 208 hospitals of the United States between 2014-2015. It records all patients, demographics, vital sign measurements, diagnosis information, and treatment information in detail [17].

### Ethical Considerations

Ethical approval and individual patient consent was not necessary because all the protected health information was anonymized.

### Study Population

All patients in the eICU-CRD database were considered. The inclusion criteria were as follows: (1) patients were diagnosed with HF according to the International Classification of Diseases, ninth and tenth Revision codes (Multimedia Appendix 1); (2) the diagnosis priority label was “primary” when admitted to the ICU in 24 hours; (3) the ICU stay was more than 1 day; and (4) patients were aged 18 years or older. Patients who had more than 30% missing values were excluded [18].

### Predictor Variables

The prediction outcome of the study is the probability of in-hospital mortality, defined as patient’s condition upon leaving the hospital. Based on previous studies [19-22] and experts’ opinion (a total of 6 independent medical professionals and cardiologists in West China Hospital of Sichuan University), demographics, comorbidities, vital signs, and laboratory findings (Multimedia Appendix 2) were extracted from the eICU-CRD, using Structured Query Language (MySQL) queries (version 5.7.33; Oracle Corporation). The following tables from eICU-CRD were used: “diagnosis,” “intakeoutput,” “lab,” “patient,” and “nursecharting.” Except for the demographic characteristics, other variables were collected during the first 24 hours of each ICU admission. Furthermore, to avoid overfitting, Least Absolute Shrinkage and Selection Operator (LASSO) is used to select and filter the variables [23,24].

### Missing Data Handling

Variables with missing data are a common occurrence in eICU-CRD. However, analyses that ignore missing data have the potential to produce biased results. Therefore, we used multiple imputation for missing data [25]. All selected variables contained <30% missing values. Data were assumed missing at random and were imputed using fully conditional specification with the “mice” package (version 3.13.0) for R (version 4.1.0; R Core Team).

### Machine Learning Explainable Tool

The interpretation of the prediction model is performed by SHAP, which is a unified approach to calculate the contribution and influence of each feature toward the final predictions precisely [26]. The SHAP values can show how much each predictor contributes, either positively or negatively, to the target variable. Besides, each observation in the data set could be interpreted by the particular set of SHAP values.

### Statistical Analysis

All statistical analysis and calculations were performed using R software and Python (version 3.8.0; Python Software Foundation). The categorical variables are expressed as total numbers and percentages, and the χ^2^ test or Fisher exact test (expected frequency <10) is used to compare the differences between groups. The continuous variables are expressed as median and IQR, and the Wilcoxon rank sum test is used when comparing the two groups.

Four machine learning models—XGBoost, logistic regression (LR), random forest (RF), and support vector machine (SVM)— were used to develop the predictive models. The prediction performance of each model was evaluated by the area under the receiver operating characteristic curve. Moreover, we calculated the accuracy, sensitivity, positive predictive values, negative predictive values, and *F*_1_ score when the prediction specificity was fixed at 85%. Additionally, to assess the utility of models for decision-making by quantifying the net benefit at different threshold probabilities, decision curve analysis (DCA) was conducted [27].

## Results

### Patient Characteristics

**Figure 1:**
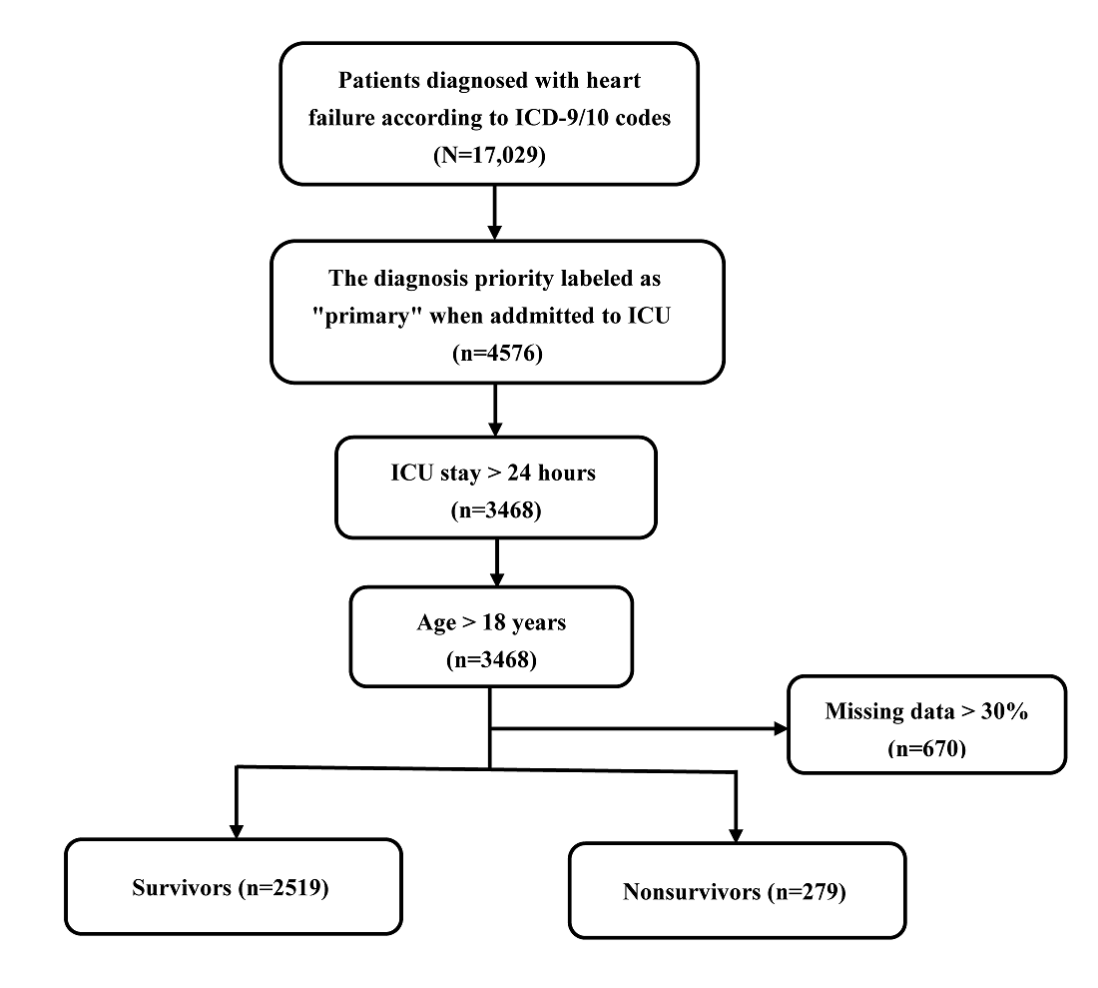
Flowchart of patient selection. ICD: International Classification of Diseases; ICU: intensive care unit.

Among 17,029 patients with HF in eICU-CRD, a total of 2798 adult patients diagnosed with primary HF were included in the final cohort for this study. The patient screening process is shown in Figure 1. The data set was randomly divided into 2 parts: 70% (n=1958) of the data were used for model training, and 30% (n=840) of the data were used for model validation. The LASSO regularization process resulted in 24 potential predictors on the basis of 1958 patients in the training data set, which were used for model developing. Patients in the nonsurvivor group were older than the ones in the survivor group (*P*<.001). The hospital mortality rate was 9.96% (195/1958) in the training data set and 10% (84/840) in the testing data set (Multimedia Appendix 3). Table 1 shows the comparisons of predictor variables between survivors and nonsurvivors during hospitalization.

**Table 1:** All predictor variables for patients with heart failure (N=2798).

			Survivors (n=2519)		Nonsurvivors (n=279)	*P* value
Age (years), median (IQR)			71 (60-80)		76 (66-82)	<.001
Gender (male), n (%)			1338 (53.1)		170 (60.9)	.02
**Comorbidities, n (%)**						
	Hypertension	654 (26)		46 (16.5)		<.001
	Acute renal failure	441 (17.5)		78 (28.0)		<.001
**Vital signs, median (IQR)**						
	Heartrate_min^a^	70 (61-80)		74 (62-86)		<.001
	Respiratory rate_avg^b^	20.1 (17.8-23.0)		21.8 (19.0-26.0)		<.001
	Respiratory rate_max^c^	27 (24-32)		32 (26-38)		<.001
	Nibp^d^_systolic_avg	120.0 (107.1-134.8)		109.0 (100.1-121.4)		<.001
	Nibp_systolic_min	95 (83-110)		84 (72-97)		<.001
	Nibp_diastolic_min	49 (41-57)		45 (35-52.5)		<.001
	Temperature_max	37 (37-37)		37 (37-38)		.03
	Temperature_min	36 (36-37)		36 (36-37)		.007
**Laboratory variables, median (IQR)**						
	Urineoutput	1550 (599-2750)		875 (140-1900)		<.001
	SpO_2_^e^_min	92 (88-95)		90 (84.5-94)		<.001
	SpO_2__avg	96.6 (95.1-98.0)		96.5 (94.5-97.9)		.04
	Anion_gap_max	11.0 (9.0-14.0)		12.0 (10.0-15.0)		<.001
	Creatinine_min	1.45 (1.01-2.30)		1.70 (1.19-2.50)		.001
	Blood_urea_nitrogen_avg	30.0 (21.0-47.6)		42.0 (28.0-58.5)		<.001
	Calcium_min	8.6 (8.1-9.0)		8.5 (7.9-8.9)		.005
	Chloride_min	101 (97-104)		99 (95-104)		.01
	Platelets×1000_min	193 (149-249)		180 (140-235.5)		.008
	White_blood_cell×1000_min	9.1 (6.8-12.1)		10.9 (7.6-15.7)		<.001
	RDW^f^_min	15.7 (14.4-17.3)		16.4 (15.0-18.2)		<.001
	Hemoglobin_max	10.6 (9.2-12.3)		10.4 (8.95-12.0)		.059

^a^Min: minimum.

^b^Avg: average.

^c^Max: maximum.

^d^Nibp: noninvasive blood pressure.

^e^SpO_2_: O_2_ saturation.

^f^RDW: red blood cell distribution width.

### Model Building and Evaluation

**Figure 2:**
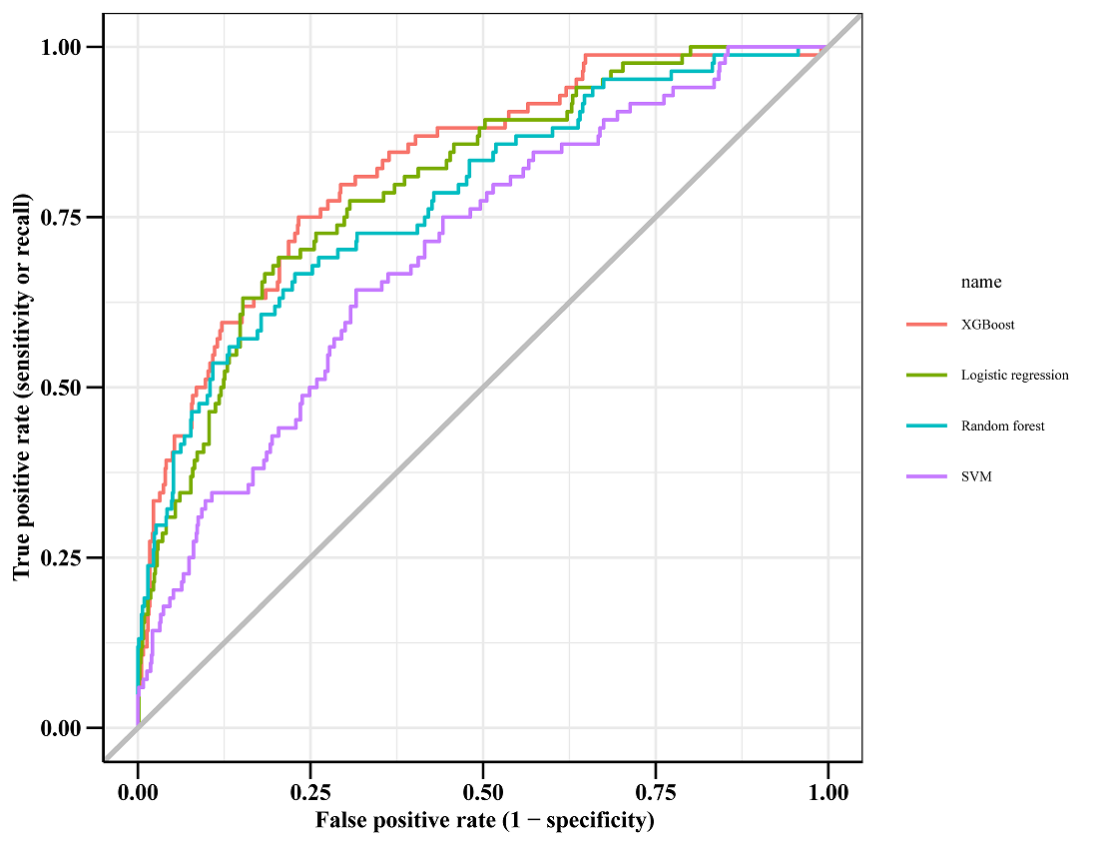
The receiver operating characteristic curve among the four models for patients with heart failure. SVM: support vector machine.

**Figure 3:**
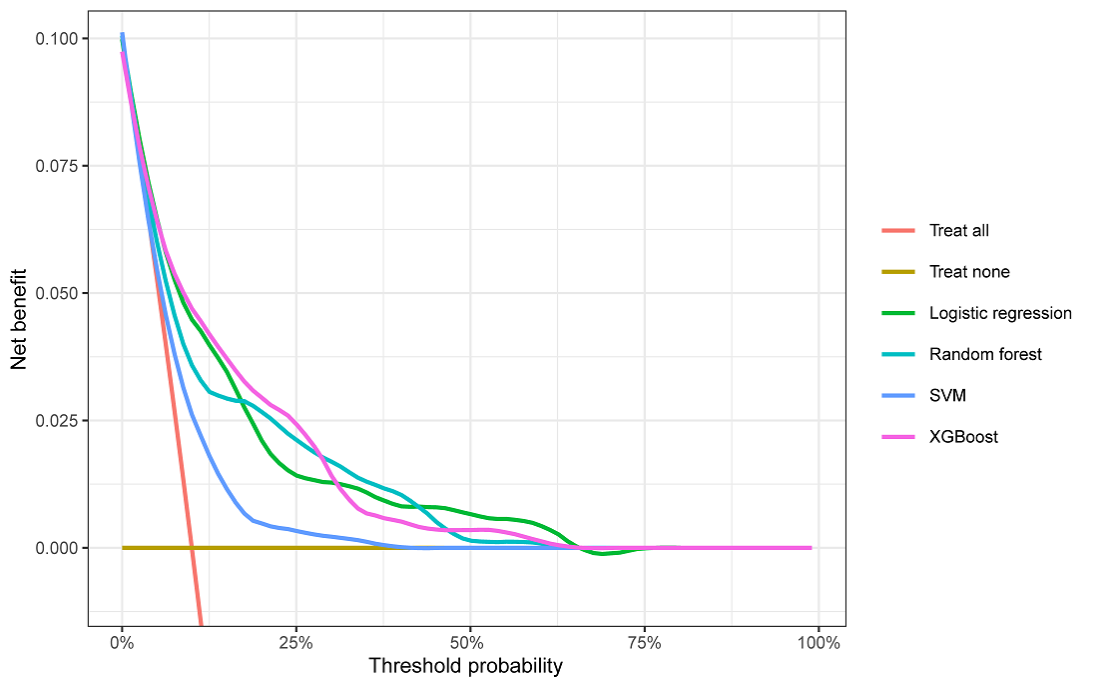
Decision curve analysis of four models plotting the net benefit at different threshold probabilities. SVM: support vector machine.

Within the training data set, the XGBoost, LR, RF, and SVM models were established, and the testing data set obtained AUCs of 0.824, 0.800, 0.779, and 0.701, respectively (Table 2 and Figure 2). Comparatively, XGBoost had the highest predictive performance among the four models (AUC=0.824, 95% CI 0.7766-0.8708), whereas SVM had the poorest generalization ability (AUC=0.701, 95% CI 0.6433-0.7582). DCA was performed for four machine learning models in the testing data set to compare the net benefit of the best model and alternative approaches for clinical decision-making. Clinical net benefit is defined as the minimum probability of disease, when further intervention would be warranted [28]. The plot measures the net benefit at different threshold probabilities. The orange line in Figure 3 represents the assumption that all patients received intervention, whereas the yellow line represents that none of the patients received intervention. Due to the heterogeneous profile of the study population, treatment strategies informed by any of the four machine learning–based models are superior to the default strategies of treating all or no patient. The net benefit of the XGBoost model surpassed those of the other machine learning models at 10%~28% threshold probabilities (Figure 3).

**Table 2:** Performance of each model for prediction.

Model	AUC^a^ (%)	Sensitivity (%)	*F*_1_ score	Accuracy (%)	PPV^b^	NPV^c^
XGBoost	0.824	0.595	0.407	0.826	0.307	0.950
LR^d^	0.800	0.607	0.413	0.827	0.311	0.951
RF^e^	0.779	0.571	0.392	0.823	0.298	0.947
SVM^f^	0.701	0.345	0.258	0.801	0.204	0.921

^a^AUC: area under the curve.

^b^PPV: positive predictive value.

^c^NPV: negative predictive value.

^d^LR: logistic regression.

^e^RF: random forest.

^f^SVM: support vector machine.

### Explanation of XGBoost Model With the SHAP Method

**Figure 4:**
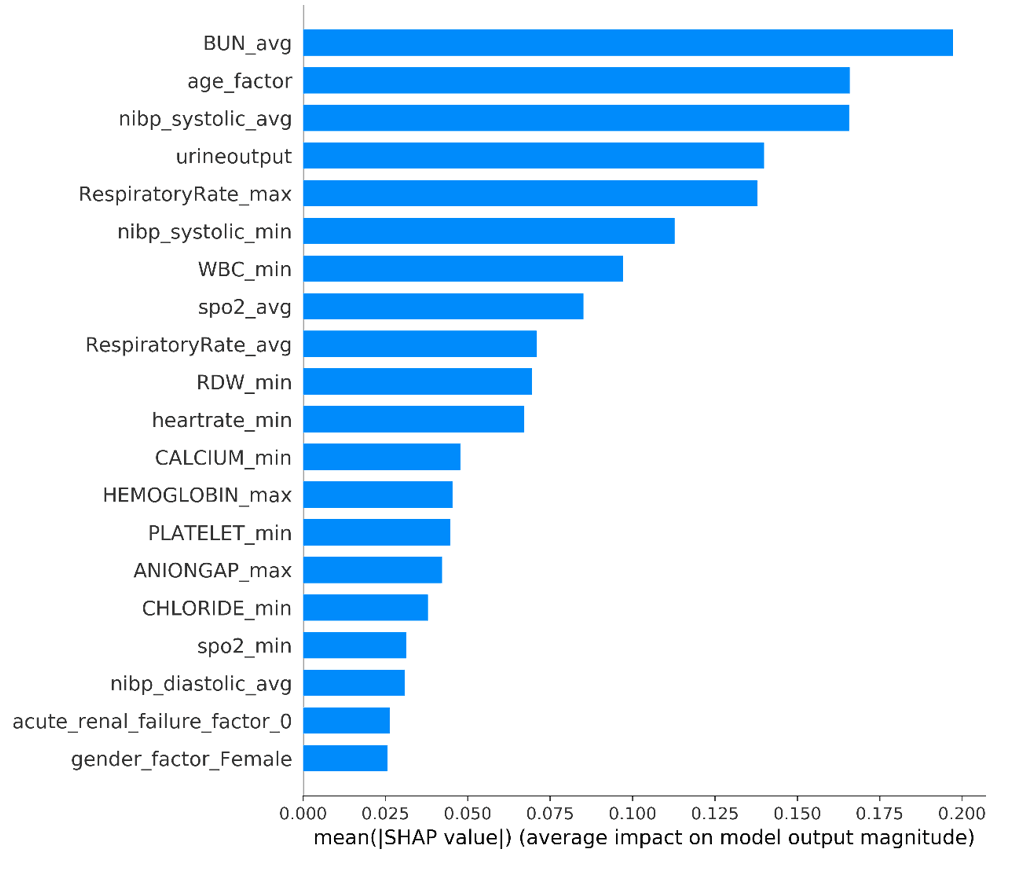
The weights of variables importance. Avg: average; BUN: blood urea nitrogen; max: maximum; min: minimum; NIBP: noninvasive blood pressure; RDW: red blood cell distribution width; SHAP: SHapley Additive exPlanation; SpO_2_: O_2_ saturation; WBC: white blood cell.

**Figure 5:**
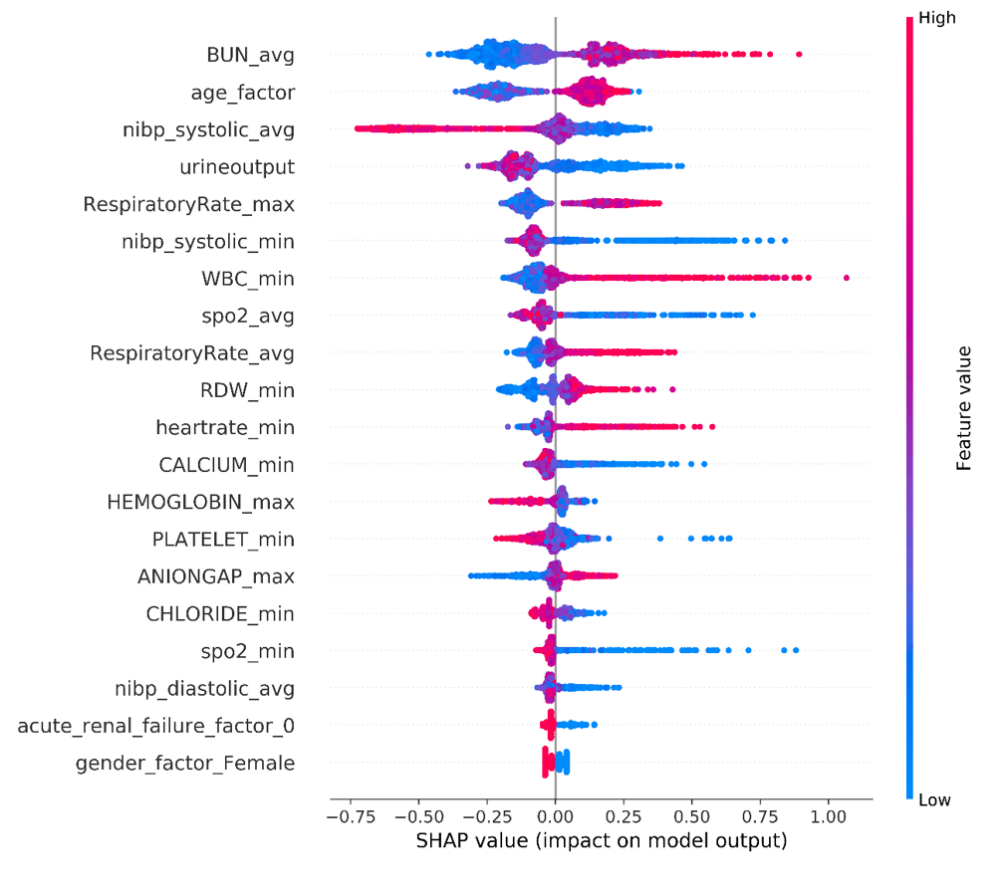
The SHapley Additive exPlanation (SHAP) values. Avg: average; BUN: blood urea nitrogen; max: maximum; min: minimum; NIBP: noninvasive blood pressure; RDW: red blood cell distribution width; SpO_2_: O_2_ saturation; WBC: white blood cell.

The SHAP algorithm was used to obtain the importance of each predictor variable to the outcome predicted by the XGBoost model. The variable importance plot lists the most significant variables in a descending order (Figure 4). The average of blood urea nitrogen (BUN) had the strongest predictive value for all prediction horizons, followed quite closely by the age factor, the average of noninvasive systolic blood pressure, urine output, and the maximum of respiratory rate. Furthermore, to detect the positive and negative relationships of the predictors with the target result, SHAP values were applied to uncover the mortality risk factors. As presented in Figure 5, the horizontal location shows whether the effect of that value is associated with a higher or lower prediction and the color shows whether that variable is high (in red) or low (in blue) for that observation; we can see that increases in the average BUN has a positive impact and push the prediction toward mortality, whereas increases in urine output has a negative impact and push the prediction toward survival.

### SHAP Individual Force Plots

**Figure 6:**
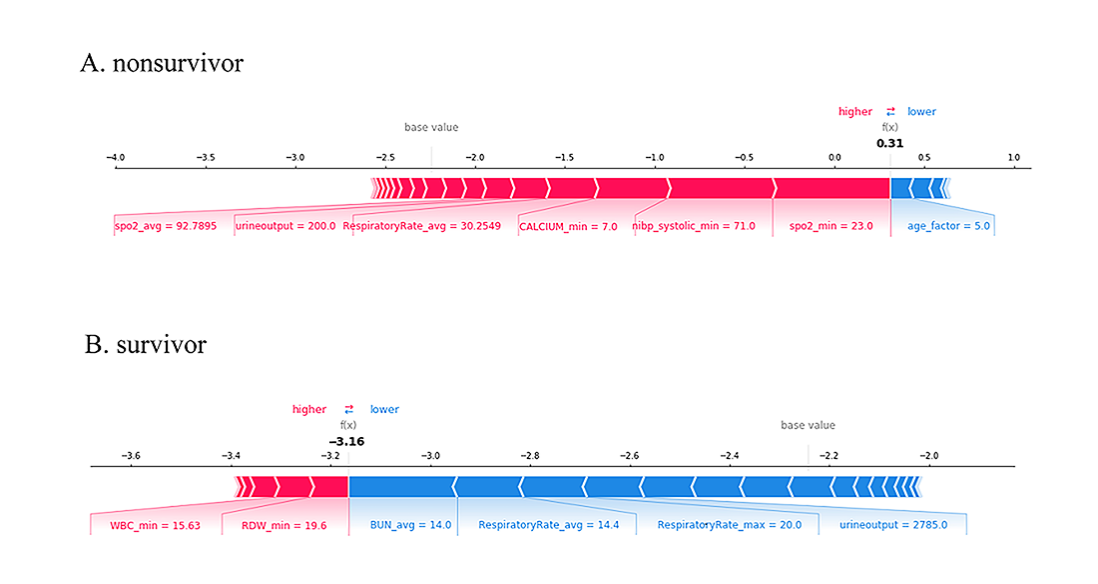
SHapley Additive exPlanation (SHAP) force plot for two selected patients.

Figure 6 shows the individual force plots for patients who (A) did not survive and (B) survived. The SHAP values indicate the prediction-related feature of individual patients and the contribution of each feature to the mortality prediction. The bold-faced numbers are the probabilistic predicted values (f(x)), whereas the base values are the values predicted without giving input to the model. The f(x) is the log odds ratio of each observation. The red features (on the left) indicate features that increase the mortality risk, and the blue features indicate features that decrease the mortality risk. The length of the arrows helps visualize the magnitude of the effect on the prediction. The longer the arrow, the larger the effect.

## Discussion

### Principal Findings

In this retrospective cohort study of a large-scale public ICU database, we developed and validated four machine learning algorithms to predict the mortality of patients with HF. The XGBoost model outperforms the performance of LR, RF, and SVM. The SHAP method is used to explain the XGBoost model, which ensures the model performance and clinical interpretability. This will help physicians better understand the decision-making process of the model and facilitates the use of prediction results. Besides, to avoid ineffective clinical interventions, the relevant threshold probability range of DCA that we focused on was between 15% and 25%, during which XGBoost performed better. In critical care research, XGBoost has been widely used to predict the in-hospital mortality of patients and may assist clinicians in decision-making [29-31]. However, the mortality of patients with HF included in the final cohort is just 9.97%. Although DCA shows that the XGBoost model is better than the two default strategies, the positive predictive value is just 0.307 when the prediction specificity is fixed at 85%. Therefore, the XGBoost model may not be fully acceptable to provide decision-making support for clinicians. Evaluation of the benefits of earlier prediction of mortality and its additional cost is necessary in clinical practice.

Using SHAP to explain the XGBoost model, we identified some important variables associated with in-hospital mortality of patients with HF. In this study, the average BUN was recognized as the most important predictor variable. As a renal function marker to measure the amount of nitrogen in blood that comes from protein metabolism, previous studies also showed that BUN was the key predictor of HF mortality prediction with machine learning algorithms [32,33]. Kazory [34] concludes that BUN may be a biomarker of neurohormonal activation in patients with HF. From the perspective of pathophysiology, the activity of sympathetic nervous systems and the renin-angiotensin-aldosterone system increases with the aggravation of HF, which causes the vasoconstriction of the afferent arterioles. A reduction in renal perfusion further leads to water and sodium retention and promotes urea reabsorption, ultimately resulting in an increased BUN. However, further research is needed to explore the applicability of this SHAP method, due to the lack of an external validation cohort.

### Limitations

This study had some limitations. First, our data were extracted from a publicly available database, and some variables were missing. For example, we intended to include more predictor variables that may affect in-hospital mortality such as prothrombin time as well as brain natriuretic peptide and lactate; however, the missing values were over 70%. Second, all data were derived from the ICU patients from the United States, so the applicability of our model remained unclear in other populations. Third, our mortality prediction models were based on data available within 24 hours of each ICU admission, which may neglect the subsequent events that change the prognosis and cause confounders to some extent. Fourth, due to lack of an external validation cohort, the applicability of the developed XGBoost model may not be very efficient in clinical practice. Currently, we are trying to collect data of patients with HF in ICUs from West China Hospital of Sichuan University. Although we have obtained some preliminary data, it is not feasible for the external prospective validation because of the limited sample size.

### Conclusions

We developed the interpretable XGBoost prediction model that has the best performance in estimating the mortality risk in patients with HF. In addition, the interpretable machine learning approach can be applied to accurately explore the risk factors of patients with HF and enhance physicians’ trust in prediction models. This will help physicians identify patients with HF who have a high mortality risk so as to timely apply appropriate treatments for them.

## Appendix

Multimedia Appendix 1 Supplementary material 1: the ICD 9/10 codes.

Multimedia Appendix 2 Supplementary material 2: the selected predictor variables.

Multimedia Appendix 3 Supplementary material 3: all predictor variables for patients in the training and testing data set.

## Abbreviations

AUC: area under the curve
BUN: blood urea nitrogen
DCA: decision curve analysis
eICU-CRD: eICU Collaborative Research Database
HF: heart failure
ICU: intensive care unit
LASSO: Least Absolute Shrinkage and Selection Operator
LR: logistic regression
RF: random forest
SHAP: SHapley Additive exPlanation
SVM: support vector machine
