# Supplementary material for: Predicting Mortality in Intensive Care Unit Patients With Heart Failure Using an Interpretable Machine Learning Model: Retrospective Cohort Study
Source: J Med Internet Res. 2022 Aug 9;24(8):e38082. doi: 10.2196/38082 (PMC9399880; doi:10.2196/38082)
Supplement: Multimedia Appendix 2 [file jmir_v24i8e38082_app2.docx]

| Predictor variables | | | |
| --- | --- | --- | --- |
| Name | Unit | Comment | |
| demographic characteristics | | | |
| age | year | age of the patient in full years |  |
| gender | / | gender of the patient: Male, Female |  |
| Body Mass Index(BMI) | kg/m2 | defined as the [body mass](https://en.wikipedia.org/wiki/Human_body_weight)(admission weight of the patient in kilograms) divided by the [square](https://en.wikipedia.org/wiki/Square_(algebra)) of the [body height](https://en.wikipedia.org/wiki/Human_height)(admission height of the patient in meters) |  |
| comorbidities | | |  |
| diabetes mellitus | Factor:0 means this patient has the disease, while 1 means the patient doesn’t. | comorbidities were identified using ICD-9/10 codes. |  |
| hypertension |  |  |  |
| atrial fibrillation |  |  |  |
| chronic kidney disease |  |  |  |
| acute renal failure |  |  |  |
| chronic obstructive pulmoriary disease(COPD) |  |  |  |
| vital signs | | |  |
| heart rate | beat per minute | These variables were collected during the first 24 hours of each ICU admission. Considering there are records of more than one time, **the minimum, maximum and average** of each variable were collected. |  |
| respiratory Rate | times per minute |  |  |
| O2 Saturation | % |  |  |
| non-Invasive BP Systolic | mmHg |  |  |
| non-Invasive BP Diastolic | mmHg |  |  |
| non-Invasive BP Mean | mmHg |  |  |
| temperature | ℃ |  |  |
| urineoutput | ml | the urine of this patient during the first 24 hours of ICU admission |  |
| laboratory variables | | |  |
| anion gap | mmol/L | These variables were collected during the first 24 hours of each ICU admission. Considering there are records of more than one time, **the minimum, maximum and average** of each variable were collected. |  |
| creatinine | mg/dL |  |  |
| blood urea nitrogen(BUN) | mg/dL |  |  |
| glucose | mg/dL |  |  |
| potassium | mmol/L |  |  |
| sodium | mmol/L |  |  |
| calcium | mg/dL |  |  |
| chloride | mmol/L |  |  |
| platelets x 1000 | K/mcL |  |  |
| white blood cell(WBC) x 1000 | K/mcL |  |  |
| red blood cell(RBC) | M/mcL |  |  |
| mean corpusular volume(MCV) | fL |  |  |
| red blood cell volume distribution width(RDW) | % |  |  |
| hematocrit(Hct) | % |  |  |
| the level of mean corpsular hemoglobin(MCH) | pg |  |  |
| mean corpuscular hemoglobin concentration | g/dL |  |  |
| hemoglobin(Hgb) | g/dL |  |  |
