# Supplementary material for: Predicting Mortality in Intensive Care Unit Patients With Heart Failure Using an Interpretable Machine Learning Model: Retrospective Cohort Study
Source: J Med Internet Res. 2022 Aug 9;24(8):e38082. doi: 10.2196/38082 (PMC9399880; doi:10.2196/38082)
Supplement: Multimedia Appendix 3 [file jmir_v24i8e38082_app3.docx]

Supplementary material 3. All predictor variables for patients with heart failure in the training and testing data set.

|  | Training Data Set | | |  | Testing Data Set | | |
| --- | --- | --- | --- | --- | --- | --- | --- |
|  | Survival(n=1763) | Nonsurvival(n=195) | P |  | Survival(n=756) | Nonsurvival(n=84) | P |
| Demographic characteristics |  |  |  |  |  |  |  |
| Age (year), median(IQR) | 71[60-80] | 75[66-82] | <.001 |  | 71[60-81] | 76[66.5-82] | .01 |
| Gender (male), n (%) | 932(52.9) | 118(60.5) | .050 |  | 406(53.7) | 52(61.9) | .19 |
| Comorbidities,n(%) |  |  |  |  |  |  |  |
| hypertension | 458(26.0) | 31(15.9) | .003 |  | 196(26.0) | 15(17.9) | .14 |
| acute renal failure | 303(17.2) | 58(29.7) | <.001 |  | 138(18.3) | 20(23.8) | .28 |
| Vital signs, median (IQR) |  |  |  |  |  |  |  |
| urineoutput | 1535[577.5,2692.5] | 875[150,2032.5] | <.001 |  | 1625[600,2832.5] | 887.5[47.5,1617.5] | <.001 |
| heartrate_min^a^ | 70[61,80] | 74[62,87] | .002 |  | 71[62,81] | 75[61.75,85] | .16 |
| RespiratoryRate_avg^b^ | 20.0[17.8,23.0 ] | 21.2[18.8,25.3] | <.001 |  | 20.3[18.0,22.8] | 23.0[19.5,26.7] | <.001 |
| RespiratoryRate_max^c^ | 27[24,32] | 30[25,37] | <.001 |  | 28[24,33] | 33[27.75,40] | <.001 |
| nibp^d^_systolic_avg | 120.0[107.3,134.8] | 110.6[101.3,123.1] | <.001 |  | 119.4[106.7,134.4] | 105.9[98.3,116.0] | <.001 |
| nibp_systolic_min | 96[83,110] | 87[74,101] | <.001 |  | 94[82,110] | 81[70,91] | <.001 |
| nibp_diastolic_min | 49[40,57] | 46[35.5,54] | <.001 |  | 48[41,56] | 42[35,49] | <.001 |
| temperature_max | 37[37,37] | 37[37,38] | .03 |  | 37[37,37] | 37[37,38] | .57 |
| temperature_min | 36[36,37] | 36[36,37] | .02 |  | 36[36,37] | 36[36,37] | .21 |
| Laboratory variables, median (IQR) |  |  |  |  |  |  |  |
| anion_gap_max | 11.0[9.00,14.0] | 12.0[10.0,15.2] | <.001 |  | 11.3[9.00,14.0] | 13.0[8.60,15.0] | .10 |
| creatinine_min | 1.44[1.01,2.29] | 1.62[1.10, 2.39 ] | .07 |  | 1.46[1.01,2.44] | 1.90[1.31,2.78] | .002 |
| spo2^e^_min | 92[88,95] | 90[84.5,94] | <.001 |  | 92[88,95] | 91[84.75,94] | .02 |
| spo2_avg | 96.5[95.1,98.0] | 96.3[94.1,97.9] | .020 |  | 96.7[95.1,98.1] | 96.8[ 95.3,98.0] | .86 |
| blood_urea_nitrogen_avg | 30.0[21.0,46.8] | 41.5[26.0,56.5] | <.001 |  | 31.0[21.0,49.1] | 44.5[31.8,62.0] | <.001 |
| calcium_min | 8.60[8.20,8.90] | 8.40[7.90,8.90] | .02 |  | 8.60[8.10,9.00] | 8.50[7.90,8.90] | .095 |
| chloride_min | 101[96.0,105] | 99.0[95.0,103.0] | .02 |  | 101[97.0,104] | 99.5[96.0,104] | .37 |
| platelets x 1000_min | 195[150,252] | 185[143,244] | .091 |  | 187[145,241] | 162[130.8,218.5] | .024 |
| white_blood_cell x 1000_min | 9.20[6.90,12.2] | 10.8[7.25,15.3] | <.001 |  | 9.00[6.70,12.0] | 11.7[8.45,16.0] | <.001 |
| RDW^f^_min | 15.7[14.4,17.3] | 16.4[14.9,18.2] | <.001 |  | 15.8[14.4,17.3] | 16.5[15.2,18.1] | .002 |
| hemoglobin_max | 10.6[9.3,12.4] | 10.3[8.90,11.9] | .031 |  | 10.5[9.10,12.2] | 10.9[9.28,12.1] | .91 |

^a^Min: minimum.

^b^Avg: average.

^c^Max: maximum.

^d^Nibp: noninvasive blood pressure.

^e^Spo2: O2 saturation.

^f^RDW: red blood cell distribution width.
